# Regulation, targets and functions of CSK

**DOI:** 10.3389/fcell.2023.1206539

**Published:** 2023-06-16

**Authors:** Shudong Zhu, Hui Wang, Kamakshi Ranjan, Dianzheng Zhang

**Affiliations:** ^1^ School of Medicine, Nantong University, Nantong, China; ^2^ Department of Bio-Medical Sciences, Philadelphia College of Osteopathic Medicine, Philadelphia, PA, United States

**Keywords:** Csk, Chk, src, SFK, activity, regulation, target

## Abstract

The Src family kinases (SFK) plays an important role in multiple signal transduction pathways. Aberrant activation of SFKs leads to diseases such as cancer, blood disorders, and bone pathologies. By phosphorylating and inactivating SFKs, the C-terminal Src kinase (CSK) serves as the key negative regulator of SFKs. Similar to Src, CSK is composed of SH3, SH2, and a catalytic kinase domain. However, while the Src kinase domain is intrinsically active, the CSK kinase domain is intrinsically inactive. Multiple lines of evidence indicate that CSK is involved in various physiological processes including DNA repair, permeability of intestinal epithelial cells (IECs), synaptic activity, astrocyte-to-neuron communication, erythropoiesis, platelet homeostasis, mast cell activation, immune and inflammation responses. As a result, dysregulation of CSK may lead to many diseases with different underlying molecular mechanisms. Furthermore, recent findings suggest that in addition to the well-established CSK-SFK axis, novel CSK-related targets and modes of CSK regulation also exist. This review focuses on the recent progress in this field for an up-to-date understanding of CSK.

## 1 Introduction

The family Src tyrosine kinase (SFKs) consist of eight members including Src, Yes, Fyn, Lyn, Lck, Fgr, Hck, and Blk. Src is the first member discovered in this family and is ubiquitously expressed in all cell types. Src has been found to play important roles in a variety of cellular processes including cell proliferation and growth. Aberrant elevation of Src kinase activity is highly related to the development of cancers (Caner et al., 2021).

Src kinase activity is regulated post-translationally by phosphorylation. Phosphorylation at the autophosphorylation site Y419 (human) makes Src adapt a configuration favoring the binding of its substrates and therefore activates Src. On the other hand, phosphorylated Y530 results in Src inactivation. Y530 of Src is phosphorylated by CSK, a ubiquitously expressed protein kinase. To counteract against CSK and activate Src, phosphorylated Y530 is dephosphorylated by several protein tyrosine phosphatases including PTP-α, PTP-λ, SHP-1, SHP-2, and PTP1B (Zhu et al., 2007; Caner et al., 2021).

In this article, we review the recent progress in this field in understanding the regulation, molecular targets, and biological functions of CSK. With the wider application of new techniques, future research in this field will not only enhance our understanding of CSK signaling but also advance its clinical applications.

## 2 Structure of CSK

Similar to Src, CSK is composed of SH3, SH2, and a catalytic kinase domain of 450 amino acids. The main structural features of catalysis and regulation have been recently illustrated by Sun, in this topic series, and Dias (Dias et al., 2022; Sun and Ayrapetov, 2023). Unlike Src, the CSK kinase domain by itself is inactive. Instead, it is activated by interactions with the SH3, SH2 domains, as well as the SH3-SH2 linker (Sondhi & Cole, 1999; Shekhtman et al., 2001). Some residues in the SH2 domain also suppresses the CSK kinase activity, but the suppression is removed by the binding of the SH2 domain to its ligand (Lin et al., 2006).

## 3 CSK family members

In addition to CSK, CSK homologous kinase (CHK) plays a similar role in phosphorylating Y530 and inactivating Src. However, the expression of CHK is specifically limited to the brain, hematopoietic and colon epithelial cells (Klages et al., 1994; Zhu et al., 2008; Zhu et al., 2021). In particular, downregulation of CHK due to epigenetic regulation contributes significantly to tumorigenicity in colon cancer (Zhu et al., 2008; Chüeh et al., 2021; Zhu et al., 2021). In addition, CHK may serve as an allosteric inhibitor of Src (Chong et al., 2004; Chong et al., 2006; Zhu et al., 2008). However, while CHK is an efficient inhibitor, compared to CSK, it is not as efficient in its protein kinase function of phosphorylating Src C-terminal tyrosine (Advani et al., 2017). CHK may also have molecular targets other than SFKs.

## 4 Molecular targets of CSK

**FIGURE 1:**
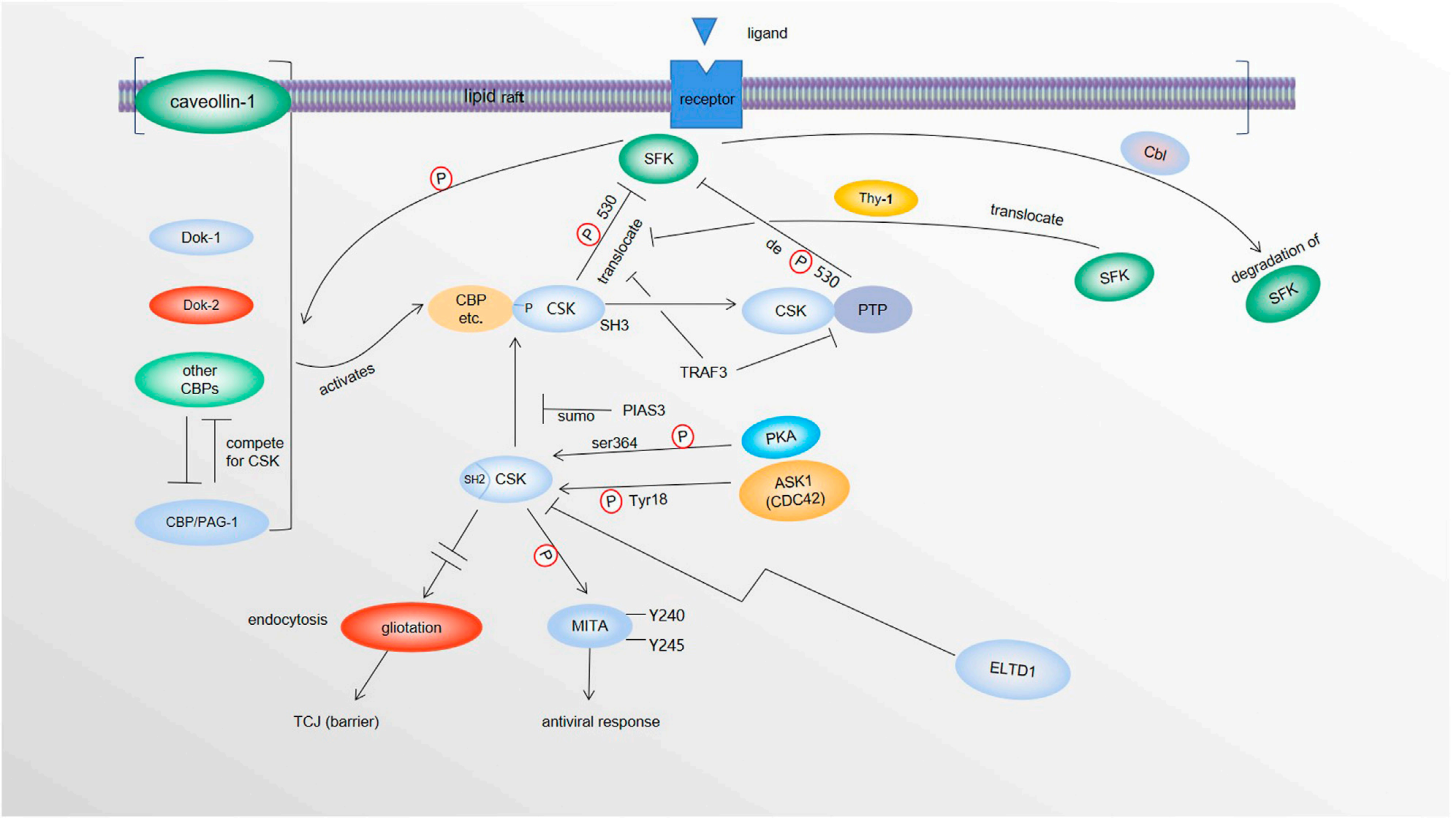
Regulation and molecular targets of CSK. SFKs in the lipid rafts phosphorylate CBP/PAG-1 and other CSK binding proteins (caveolin-1, Dok-1, Dok-2, etc.), which activate and recruit CSK (via SH2 of CSK) to the lipid rafts where SFKs are enriched. CSK phosphorylates SFKs at its Y530, causing inactivation of SFKs. By recruiting protein tyrosine phosphatases (PTPs) via its SH3 domain, CSK also recruits the phosphatases to dephosphorylate and activate SFKs. Activated SFKs can be degraded by Cbl-family mediated-ubiquitination and degradation. SFKs are also likely brought closer to CBP/CSK complex by Thy-1 to be inactivated. Both TRAF3 and PIAS3 negatively modulate the tumor suppressor function of CSK. TRAF3 dissociates CSK and PTP from SFKs. PIAS3 reduces CSK/CBP interaction by SUMOylating CSK. PKA phosphorylates CSK at Ser364 to make CSK more kinase active. CDC42 kinase 1 (ACK1) also activates CSK via phosphorylation of CSK at Y18. ELTD1 can interact with and lower the level of CSK. Upon DNA virus infection, CSK phosphorylates MITA at its Y240 and Y245, to activate MITA to promote innate antiviral response. When CSK levels are elevated, via a Src-independent mechanism(s), overexpression of tricellular junction (TCJ) protein gliotactin are decreased by increased endocytosis of gliotactin. Loss of gliotactin usually results in breakdown of the TCJs, leading to the loss of the barrier function.

The effects of CSK on SFKs is largely dependent on the CSK binding protein CBP/PAG-1, which uses its phosphotyrosine-containing motif to bind the SH2 domain of CSK. This binding not only activates CSK (Kawabuchi et al., 2000; Takeuchi et al., 2000; Lin et al., 2005; Matsubara et al., 2010), but also recruits CSK to SFK enriched lipid rafts (Yasuda et al., 2002; Xu et al., 2005; Matsubara et al., 2010; Kanou et al., 2011). This makes CBP an essential player in CSK-mediated phosphorylation/inactivation of SFKs (Figure 1). In immune cells, CSK binds and recruits protein tyrosine phosphatases such as PTPN12 (PTP-PEST) (Davidson et al., 1997; Ghose et al., 2001) or PTPN22 (LYP/Pep) (Cloutier & Veillette, 1996; Gregorieff et al., 1998) to dephosphorylate and activate SFKs via its SH3 domain. When the T cells are stimulated by CD3/CD28, TRAF3 downregulates membrane-bound CSK and PTPN22 to suppress the inhibitory phosphorylation of C-terminal Y505 and enhance activational dephosphorylation of Y394 to ultimately activate Lck in the TCR signaling. In addition, reduced activity of CSK leads to SFK activation, which can then lead to its degradation by Cbl-family mediated-ubiquitination/degradation, a compensatory mechanism for SFK activation. Affected SFKs include 56 kDa LynA, 53 kDa LynB, Src, and Fyn, but not Yes. Of note, degradation of the SFK family member LynA is due to c-Cbl induction not absence of CSK (Kuga et al., 2020). Furthermore*,* Src is likely to be brought closer to CSK-binding protein (CBP)/CSK complex by Thy-1 to be inactivated by CSK (Maldonado et al., 2017). It has been reported that upon DNA virus infection, CSK phosphorylates MITA at its Y240 and Y245 to activate MITA and promote innate antiviral response (Figure 1) (Gao et al., 2020). Nevertheless*,* since certain CSK functions appear to be independent of Src, some unidentified targets of Src likely exist (Samarasekera and Auld, 2018).

## 5 Regulation of CSK

Unlike its structural homologue Src, CSK lacks both the Y419 autophosphorylation site and the Y530 inhibitory phosphorylation site. Therefore, CSK is not subject to the main regulatory mechanisms controlling Src activation. In fact, CSK has its own unique regulations. Phosphorylated CBP/PAG-1 not only directly activates CSK (Kawabuchi et al., 2000; Takeuchi et al., 2000; Lin et al., 2005; Matsubara et al., 2010), but also translocates CSK to the lipid rafts to target SFK (Yasuda et al., 2002; Xu et al., 2005). In addition, several other adaptor proteins such as Dok-1 and Dok-2, when phosphorylated by SFKs, may be able to recruit CSK to the plasma membrane where active SFKs are localized to suppress SFK activity as a negative feedback mechanism (Davidson et al., 2016). In mast cells, results from PAG knockout suggests that CSK binding proteins other than PAG-1 exist. Interestingly, since these binding factors may compete with each other, CSK could interact with other adaptors more efficiently and inhibit the corresponding SFK signaling more potently in the absence of PAG-1 (Potuckova et al., 2018).

Lipid raft adaptor protein caveolin-1 (Cav-1) also plays a similar role to CBP/PAG-1 in recruiting cytosolic CSK to lipid rafts (Cao et al., 2002). Upregulated Caveolin-1 interacts with CSK to enhance CSK interaction with LCK and subsequently increase phosphorylation/inactivation of LCK (Sripada et al., 2021). In addition to being an adaptor bridge, SPOP also activates CSK through binding the SPOP binding motif (SBM) in the CSK activation loop (Tawaratsumida et al., 2022). For TCR signaling pathway, interactions between TRAF3 and CSK promotes CSK dissociation from T cell membranes to repress Lck signaling (Wallis et al., 2017).

It has also been reported that PKA-mediated phosphorylation of CSK at Ser364 makes the kinase domain interact with the CSK SH3 domain more efficiently and further enhances the activity of CSK (Yaqub et al., 2003). Similar to Src, CSK can be sumoylated by SUMO1 at lysine 53 physiologically. PIAS3 has been identified as the main E3 ligase for CSK sumoylation (Cui et al., 2019). Meanwhile, phosphorylation at Ser364, which activates CSK, has no effect on its sumoylation. These results demonstrate that sumoylation of CSK negatively modulates its tumor suppressor function by reducing its interaction with CBP and therefore causing Src activation (Cui et al., 2019). ELTD1 also can interact with and lower the level of CSK which leads to Src activation and MAPK activation (Sun and Zhong, 2021).

More recent findings indicate that CDC42 kinase 1 (ACK1)-mediated phosphorylation of CSK at Y18 is involved in repression of T-cell activation. Results from both genetic and pharmacological intervention of Ack1 indicate that the ACK1/pY18/CSK axis is involved in dampening T-cell priming and T-cell trafficking to the tumor microenvironment partially responsible for the resistance to the immune checkpoint blockade (ICB) therapies. More importantly, the results of experimental inhibition of ACK1 points at ACK1’s ability to reactivate immune response in ICB-resistant tumors (Sridaran et al., 2022) (Figure 1). Additionally, it is worth noticing that CSK can form homodimers in cells (Brian et al., 2022)*,* even though it is unclear when the homodimers are formed and what the downstream effect of dimerization is.

## 6 Drugs targeting CSK

It has been reported recently that caffeic acid phenethyl ester (CAPE), a propolis component from bee hives, and its derivatives can inhibit hepatocellular carcinoma. Further experiments demonstrate that these compounds can affect both CSK and EGFR. However, it is yet to be determined whether the anticancer activities of these compounds are dependent on CSK or EGFR pathways (Liu et al., 2021). Searching a dataset of approximately four thousand compounds with combined fingerprint-based similarity search and kernel-based partial least squares analysis to predict CSK inhibitors appears to be an attractive approach to identifying potential drugs against CSK (Deokar et al., 2023).

## 7 Functions

### 7.1 CSK in regulating intestinal epithelial barrier and protecting colitis

**FIGURE 2:**
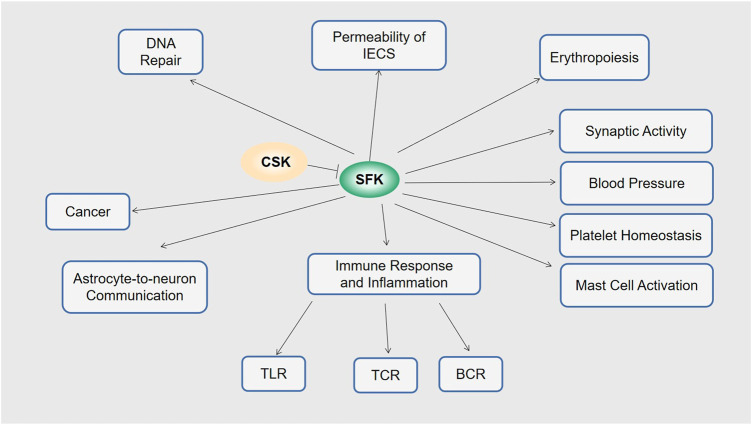
Functions of CSK. Via SFK, CSK is involved in permeability of IECs, erythropoiesis, synaptic activity, blood pressure, platelet homeostasis, mast cell activation, immune response and inflammation including TLR, TCR and BCR astrocyte-to-neuron communication, DNA repair, and cancer.

The intestinal epithelium not only allows the flow of fluids and absorption of nutrients from the intestine, but also provides a physical barrier against the intestinal pathogens. The functional barrier is maintained by intercellular adhesions and a balanced amount of epithelial cell proliferation and death. Dysfunctions of the intestinal epithelial cells (IECs) will disrupt the barrier and can result in gastrointestinal diseases such as colitis and colorectal cancer (Odenwald and Turner, 2017). In a model of inflammatory bowel disease based on dextran sodium sulfate (DSS) induction, increased susceptibility to colitis has been shown in IEC-specific CSK-deficient mice. This was associated with increased intestinal permeability accompanied by a decrease in the tight junction protein occludin, which regulates intercellular adhesion and permeability. The colon epithelial cells in this model also exhibited increased proliferation and apoptosis. These results suggest that CSKs in IECs play pivotal roles in performing intercellular barrier function and preventing colitis (Sun et al., 2018). Mechanistically, this CSK-dependent pathogenesis of DSS-induced colitis involves activation of Src in the absence of CSK, which followed by the phosphorylation and subsequent proteasomal degradation of occludin, leads to the increased intercellular permeability of the IECs (Samak et al., 2015; Imada et al., 2016; Chelakkot et al., 2017) (Figure 2).

CSK also regulates the intercellular barrier and cell permeability through regulating tricellular junction (TCJ) protein gliotactin. When CSK levels are elevated, overexpression of tricellular junction (TCJ) protein gliotactin is prevented by increased endocytosis of gliotactin. Loss of gliotactin usually results in breakdown of the TCJs, which then leads to the loss of the barrier function. On the contrary, when CSK levels are decreased, gliotactin is overexpressed which leads it to spread away from the TCJs and disrupt the epithelial architecture (Samarasekera and Auld, 2018). Therefore, the localization and the concentration of gliotactin are expected to be precisely controlled to ensure normal function of the cell barrier and epithelium. Interestingly, this CSK effect is independent of Src kinase activity.

### 7.2 CSK in cancer

With CSK as the major negative regulator of SFKs, one would assume CSK downregulation plays an important role in activation of SFKs in most cancer cells. However, evidence suggested otherwise. For example, both the protein levels and the kinase activity of CSK were abundant in breast cancer samples and breast cancer cell lines, and they exhibited no significant variations when compared to the CSK levels and kinase activity in normal human breast tissues although Src is activated in the cancer samples (Bougeret et al., 2001). Similarly, there is no significant change in CSK expression between cancerous colon tissues/cell lines and normal colon tissues/cell lines although Src is activated in the colon cancer samples with reduced phosphorylation at Src Y530 (Zhu et al., 2008; Zhu et al., 2011). Further study shows that downregulation of CSK either by a small molecule inhibitor or by shRNA knockdown does not affect SFK kinase activity or oncogenic activity in colon cancer cells. This suggests that levels of CSK are uncoupled from SFK activity in colon cancer cells. On the contrary, mis-localization of CSK could activate SFKs and lead to increased oncogenicity in colon cancer cells (Sirvent et al., 2010). There is evidence to further suggest that the functional CSK affected phosphorylation of FAK and paxillin, cell scattering, focal contacts, actin cytoskeleton rearrangement, cell adhesion, migration and invasiveness, and all these events are dependent on integrin-mediated cell adhesion (Rengifo-Cam et al., 2004). Lastly, a functional genomics screen with human prostate cancer cells identified a subclass of cancer in which simple downregulation of CSK levels affected oncogenic properties of the cells. This was reflected in the change in Src kinase activity and the promotion of castration-resistant prostate cancer (Yang et al., 2015).

A recent progress in this field has been made with ELTD1. It has been found that ELTD1 is not only significantly elevated in gastric cancer cells, but also plays a pivotal role in tumor growth and metastasis of gastric cancer due to its involvement in proliferation, migration, invasion, and epithelial-mesenchymal transition. Mechanistically, by interacting with and downregulating CSK, elevated ELTD1 leads to activation of both Src and MAPK (Sun and Zhong, 2021). Bioinformatic analysis found that high ELTD1 levels combing low CSK levels gives the best predicting performance (Sun and Zhong, 2021).

### 7.3 CSK in synaptic activity

Dopamine is a neurotransmitter and it plays important roles in the retina in light-induced synaptic processes. In retinal neurons, the canonical D1R/adenylyl cyclase/cAMP/PKA signaling pathway is activated when the dopamine D1 receptor (D1R) is stimulated by dopamine. Activated PKA leads to CSK activation and Src inhibition with subsequent downregulation of phosphorylated NMDA-type glutamate receptor (NMDAR) subunit GluN2B. This ultimately attenuates NMDA-gated currents and prevents NMDA-elicited calcium mobilization (Socodato et al., 2017).

### 7.4 CSK in astrocyte-to-neuron communication

αvβ3 integrin and its receptor Thy-1 are essential in astrocyte/neuron communication. Upon binding of αvβ3 integrin, Thy-1 forms a large Src-containing complex. Src is then inactivated by the Thy-1/CSK-binding protein (CBP)/CSK complex and removed from the complex. Inactivation of Src reduces phosphorylation of p190Rho GTPase activating protein, enhances RhoA activation, and causes phosphorylation of cofilin and myosin light chain II which eventually leads to neurite shortening. Therefore, it is likely that the membrane-bound integrins serve as trans acting ligands in astrocyte-neuron communication, and the Thy-1-CBP-CSK-Src-RhoA-ROCK axis transmits signals from astrocytic integrin-engaged Thy-1 to the actin cytoskeleton of neurons (Maldonado et al., 2017).

### 7.5 CSK in erythropoiesis

Erythropoiesis is mainly controlled by the erythropoietin (Epo) receptor signaling pathway with two key kinases Janus kinase 2 (JAK2) and Lyn. Through CSK-binding protein (CBP), CSK negatively regulates Lyn negatively to ensure proper generation of erythrocytes (Plani-Lam et al., 2017).

### 7.6 CSK in platelet homeostasis

Following blood vessel injury, platelets adhere to exposed extracellular matrix and form thrombi that transiently occlude the blood vessel to prevent blood loss. Activation of SFKs is essential for the signal transduction in this process. In mice with CSK knockout, activated platelets demonstrated increased levels of SFK although both bleeding and reduced thrombosis were also observed. This is due to several negative feedback mechanisms including downregulation of (hemi-) ITAM-containing receptors GPVI-FcR γ-chain and CLEC-2 and upregulation of the inhibitory ITIM-containing receptor G6b-B followed by activation of Shp1 and Shp2. These processes make platelets respond less dramatically to vascular injury and prevents pathological thrombosis (Mori et al., 2018). The C-terminal tyrosine of SFKs phosphorylated by CSK is dephosphorylated by CD148 in platelets and has an anti-thrombotic effect (Mori et al., 2012). Therefore, the platelet homeostasis is maintained by the counter effects of CD148 and CSK (Mori et al., 2018). CSK also appears to be the dominant inhibitor in platelets while CHK plays an auxiliary role (Nagy et al., 2020).

### 7.7 CSK in regulating blood pressure

Despite the fact that hypertension is a polygenic trait, very few associations have been made between a genetic change and the related pathways that lead to hypertension. CSK is a gene whose expression has been reported to regulate blood pressure (Lee et al., 2016). With CSK knockout mice as the model system, the mechanism of how CSK regulates blood pressure has been studied. The lack of CSK activates Src and promotes production of aldosterone in the adrenal glands by upregulation of Cyp11b2 (aldosterone synthase). Subsequently, aldosterone upregulates Sgk1 and Na+/K+-ATPase (NKA), enhancing sodium reabsorption which leads to increased plasma volume and ultimately results in hypertension (Kim et al., 2017).

### 7.8 CSK in activation of human T cell and T-cell receptor (TCR) therapeutics

SFKs positively regulate TCR signaling in naïve T cells. A balanced TCR signal depends on the opposing actions of CD45 and CSK on the C-terminal tyrosine of SFKs. By recruiting CSK to lipid rafts where Src is enriched, CBP reduces T cell growth/proliferation and enhances T cell apoptosis. Meanwhile, CBP is synergistically strengthened by CD59 (Cong et al., 2018)*.* The detailed molecular mechanism in CSK-mediated T cell apoptosis is described in detail by Fortner et al., 2022.

T cell effector function depends on TNF receptor associated factor 3 (TRAF3). Upon activation, TRAF3 forms a CD3/CD28-containing complex that dissociates CSK and PTPN22 from Lck. Since both PTPN22 and CSK are negative regulators of Lck, this dissociation leads to Lck activation and initiation of TCR signaling (Wallis et al., 2017).

In addition, Sprouty2 positively regulates T cell function via CSK. Using Spry2 knockout mouse to target T cells, it has been found that Spry2 positively regulates MAPK signaling by activating LCK, resulting in the inability of Spry2−/− CD4^+^ T cells to proliferate, differentiate into T helper cells, and produce cytokines. Mechanistically, Spry2 deficiency leads to the upregulation of lipid raft protein caveolin-1 which interacts with CSK and strengthens its interaction with LCK. This explains why people with Spry2-deficient cells have weakened human T cell proliferation and cytokine production that often manifests as airway inflammation (Sripada et al., 2021).

On the other hand, TCR-engineered T cells are a powerful tool to make the immune system work against tumors. Although TCR recognition is supposed to target the specific peptide-MHC complex, the possibility of off-target effects due to the cross-reactivity against epitopes in unintended targets still represents a severe complication for cancer patients (Hinrichs and Restifo, 2013). However, co-expression of TCR with CSK does not compromise target recognition/binding and instead makes T cells incapable of executing their effector functions. Therefore, manipulating the level of CSK in TCR-engineered T cells improves the safety of TCR therapies. These redirected, seemingly dummy T cells have now been proposed to be used as validation before clinical trials of TCR therapeutics (Inderberg et al., 2018).

### 7.9 CSK in activation of human B cell and B-cell receptor (BCR) therapeutics

Similar to TCR signaling, B cell antigen receptor (BCR) signaling is also regulated by SFKs. However, SFKs regulate BCR signaling both positively and negatively in naïve B cells. Using a PP1-analog-sensitive CSK (CSK AS) system, it has been shown that although CSK inhibition causes SFK activation in B cells, it also suppresses BCR-mediated production of phosphatidylinositol 3,4,5-trisphosphate (PIP3) in B cells, leading to marked suppression of BCR-mediated cytoplasmic calcium increase and MAPK activation. This is specifically accomplished by phosphorylated ITIM receptors (CD22, receptor adaptor Dok-1, and FcgRIIB to a lesser extent) that recruit SH2 domain-containing protein (SHPs) and lipid phosphatases like SHIP1 that directly dephosphorylate various positive signaling molecules (Lu et al., 2021). This reveals the important role of CSK in maintaining a proper level of SFK activation in quiescent B cells for tolerance of self-antigens (Lu et al., 2021). The different effects of CSK in T and B cells may have potentially varying therapeutic implications. For instance, in B cell lymphoma, use of CSK inhibitor could facilitate the T cell response against tumor and suppress the growth of transformed B cells by blocking their downstream PI3K signaling (Lu et al., 2021).

### 7.10 CSK in other immune responses

#### 7.10.1 Immune responses involving TLRs

Toll-like receptors (TLRs) are key regulators of inflammation and play indispensable roles in initiating immune responses via the adaptor protein MyD88, followed by activation of signaling pathways of NF-kB, MAPK and interferon regulatory transcription factors (IRFs). IRFs control key effector functions, including cytokine release (IL-12, etc.), cell differentiation, and sometimes inflammation pathology. IRF activity is controlled by the SFK member LYN, which phosphorylates IRFs, resulting in K48-linked polyubiquitination and proteasomal degradation of IRFs causing downregulation of nuclear IRFs. On the other hand, LYN activity is controlled by CSK, which is controlled by the TLR adaptor SPOP, a MyD88-interacting protein which helps to recruit CSK into the TLR signaling complex to exert CSK catalytic activity (Tawaratsumida et al., 2022).

#### 7.10.2 Positive and negative regulatory roles of CSK in FcεRI-Mediated mast cell activation

In mast cells, CSK positively regulates FcɛRI-induced production of proinflammatory cytokines and chemokines through the LYN/SHP-1/STAT5 axis. In contrast, CSK negatively regulates calcium response, degranulation, and chemotaxis. The calcium response was through CSK-LYN-SYK-LAT-PLCγ axis or complementary FYN-dependent pathway. Rapid Ca2+ mobilization induced mast cell degranulation. Chemotaxis involves CSK-integrin axis (Potuckova et al., 2018).

#### 7.10.3 CSK promotes innate immune response to DNA virus

The cyclic GMP-AMP (cGAMP) synthase (cGAS) in the cytoplasm senses entry of viral DNA and starts to synthesize the 2′3′cyclic GMP-AMP (cGAMP), a second messenger molecule, which binds to the protein Mediator of IRF3 Activation (MITA, also known as STING, etc.), an ER-associated membrane adaptor protein, to transduce innate antiviral response. This antiviral response is dependent on CSK (Gao et al., 2020). After the virus infects the cell, CSK phosphorylates MITA at Y240 and Y245, which is important for MITA protein stability and cGAMP binding. This phosphorylation step proves to be important for MITA activation and MITA mediated innate antiviral response (Xia et al., 2019; Gao et al., 2020). These results suggest that CSK modulates innate immune response to DNA virus.

### 7.11 CSK in DNA repair

In multidrug-resistant (MDR) cells, P-glycoprotein (P-gp) reduces DNA repair efficiency (DNA interstrand cross-linking agent (ICL)-induced). The CBP signaling pathway involved in the negative control of Src activation is enhanced in MDR cells. Further research shows two underlying mechanisms. On the one hand, P-gp downregulates DNA damage response regulators ATM, Chk2, Braca1, and Nbs1, causing inhibition of DNA double-strand break repair of cells in the presence of ICL agents. On the other hand, P-gp interacts with CBP and Src and enhance the formation of CBP-CSK-Src complexes causing reduced Src activity and leading to decreased DNA repair activity and increased susceptibility of cells to ICL agents. Therefore, the DNA ICL agents could have therapeutic potential against MDR cells overexpressing P-gp (Lin et al., 2017).

## 8 Concluding remarks

Recent progresses in CSK research related to its molecular targets, regulation, and biological roles have been discussed in this review. Although CSK has been studied more extensively than its family homologue CHK, the detailed molecular mechanisms underlying CSK-related physiology and pathology of many diseases are still largely unknown and warrants further exploration on CSK.

It is interesting that MITA has been reported as a substrate of CSK. Although Gao’s report is the only report about this, the results of MITA/CSK association, the ability of CSK to phosphorylate MITA both *in vivo* and *in vitro*, and the mutation functional assay altogether have established MITA as the direct target of CSK and its role in antiviral response. Considering that both Src and CSK kinases phosphorylate Y245 of MITA, it would be interesting to find out how Src and CSK collaborate in regulating MITA functions (Xia et al., 2019). Whether there are other novel CSK substrates, as implied in the control of CSK over gliotactin (either endocytosis of gliotactin, or spreading of gliotactin at TCJs), also deserves further study. As more than one novel substrate has recently been identified for CHK (reviewed in Zhu et al., 2022), it would not be surprising if more novel substrates are identified for its homologue CSK. Finally, the complementary role between CSK and CHK in various physiological process deserves further investigation.

It is surprising that quite a few proteins have been recently identified as regulators or potential regulators for CSK in addition to CBP/PAG-1. These include Dok-1, Dok-2, caveolin-1, SPOP, TRAF3, PKA, PIAS3, ELTD1, ASK1(CDC42). Possibly the action of CSK homodimerization is also involved in regulating CSK activity (Brian et al., 2022). It would be interesting to find out how exactly so many regulators collaborate in such a complex regulatory system.

Insufficient CSK expression has been thought to be likely an important contributor to tumorigenicity due to the ability of CSK to inactivate SFKs. However, change of CSK protein levels has not been found in many cancers, such as colon cancer (Zhu et al., 2008). Considering that CSK subcellular localization contributes to its local kinase activity and CSK recruitment to the lipid rafts is a typical process assisted by numerous adaptor recruiting regulators, it is likely that the localization of CSK, as well as changes in regulator levels (such as CBP) could be important contributors to the tumorigenicity of many types of cancers. Therefore CSK-centered therapies or diagnostics should also be multi-faceted.
